# The developmental hierarchy and scarcity of replicative slender trypanosomes in blood challenges their role in infection maintenance

**DOI:** 10.1073/pnas.2306848120

**Published:** 2023-10-12

**Authors:** Stephen D. Larcombe, Emma M. Briggs, Nick Savill, Balazs Szoor, Keith R. Matthews

**Affiliations:** ^a^School of Biological Sciences, Ashworth laboratories, Institute for Immunology and Infection Research, University of Edinburgh, Edinburgh EH9 3FL, United Kingdom; ^b^College of Medical, Veterinary and Life Sciences, School of Infection and Immunity, Wellcome Centre for Integrative Parasitology, University of Glasgow, Glasgow G12 8TA, United Kingdom

**Keywords:** parasite, *Trypanosoma brucei*, differentiation, single cell, commitment

## Abstract

The development of *Trypanosoma brucei* in its mammalian host is marked by a distinct morphological change as replicative “slender” forms differentiate into cell cycle arrested “stumpy” forms in a quorum-sensing-dependent manner. Although stumpy forms dominate chronic infections at the population level, the proportion of replicative parasites at the individual cell level and the irreversibility of arrest in the bloodstream are unclear. Here, we experimentally demonstrate that developmental cell cycle arrest is definitively irreversible in acute and chronic infections in mice. Furthermore, analysis of replicative capacity and single-cell transcriptome profiling reveal a temporal hierarchy, whereby cell cycle arrest and appearance of a reversible stumpy-like transcriptome precede irreversible commitment and morphological change. Unexpectedly, we show that proliferating parasites are exceptionally scarce in the blood after infections are established. This challenges the ability of bloodstream trypanosomes to sustain infection by proliferation or antigenic variation, these parasites instead being overwhelmingly adapted for transmission.

## Significance

African trypanosomes cause important human and animal disease in sub-Saharan Africa. The parasites sustain infections through replication as slender forms and by immune evasion through antigenic variation. By a quorum-sensing-dependent process, slender forms develop to stumpy forms that are adapted for transmission by tsetse flies. Here, we establish the hierarchy of developmental commitment between slender and stumpy forms, demonstrating that cell cycle arrest and transcriptome adaptation precede morphological change. We also demonstrate, at the single-cell level, that slender cells that are able to replicate form an exceptionally small proportion of the bloodstream population. This challenges the long-held paradigm that bloodstream trypanosomes sustain the infection through replication and antigenic variation.

African trypanosomes are protozoan parasites that cause sleeping sickness in humans and “nagana” in livestock, responsible for a significant humanitarian and economic burden in sub-Saharan Africa (1). These species mostly require a tsetse fly vector to complete their life cycles, necessitating a suite of complex developmental changes (2). Atypically among the trypanosome group, the development of *Trypanosoma brucei* in its mammalian hosts is accompanied by a distinct morphological change; replicative “slender” forms establish the infection, before differentiating into nondividing “stumpy” forms through quorum sensing (3). These stumpy forms are cell cycle arrested, more resistant to destruction by the host immune system (4), and exhibit molecular preadaptation for their onward development in tsetse flies (5). This morphological transition has been noted since the earliest descriptions of these parasites (3, 6), and much recent work has focused on uncovering the molecular signaling events that underpin the developmental changes (7). Nonetheless, fundamental questions about the commitment to differentiation of bloodstream forms in the mammalian host and the contribution of different developmental forms to the infection dynamic remain.

In particular, a trade-off is thought to exist between the parasites’ investment in slender and stumpy forms. Specifically, the proliferation of slender forms maintains the infection and promotes immune evasion by enabling the generation and expansion of antigenic variants in the parasite population (8). In contrast, the generation of stumpy forms limits the parasitemia and so promotes host survival, whereas their molecular adaptations prepare them for their transmission to tsetse (9). Being nonproliferative and uniformly arrested in their cell cycle in G1/G0, stumpy forms of *T. bruce*i have been considered terminally arrested and destined to be eliminated unless taken up by a tsetse fly, where they can resume proliferation as differentiating procyclic forms (10). This combination of immune evasion by antigenic variation, the proliferation of slender forms and the accumulation, and then senescence or immune elimination of arrested stumpy forms has long been associated with a classic profile of undulating waves of trypanosome parasitemias. However, despite long-standing acceptance that, once initiated, the transition from slender to stumpy form is terminal, evidence for this is indirect (11–17). Moreover, trypanosome parasitemias are pleomorphic, reflecting that a progressive spectrum of morphologies exists between slender and stumpy extremes, this being supported by recent single-cell transcriptomic analyses (18). Hence the relationship between cell proliferation, morphological and molecular adaption, and the reversibility of cell cycle arrest for bloodstream parasites is unclear. Added to this complexity, most experimental studies of slender and stumpy forms have focused only on the first wave of infection, whereas chronic infections are more representative of clinical trypanosomiasis in humans and animals (19).

Here, using an ex vivo assay of parasite proliferation using wild-type and quorum-sensing defective parasites, and single-cell transcriptomic analysis, we have quantitatively analyzed the proportion and molecular characteristics of replicable or arrested parasites at acute and chronic stages of infection, as well as parasites transitional between the morphological extremes. Surprisingly, our data suggest that regardless of morphology, the bloodstream parasite population has an insignificant role in the maintenance of the infection, instead being devoted to transmission.

## Results

### Bloodstream Trypanosomes Lose Replicative Competence Soon after infection.

**Fig. 1.**
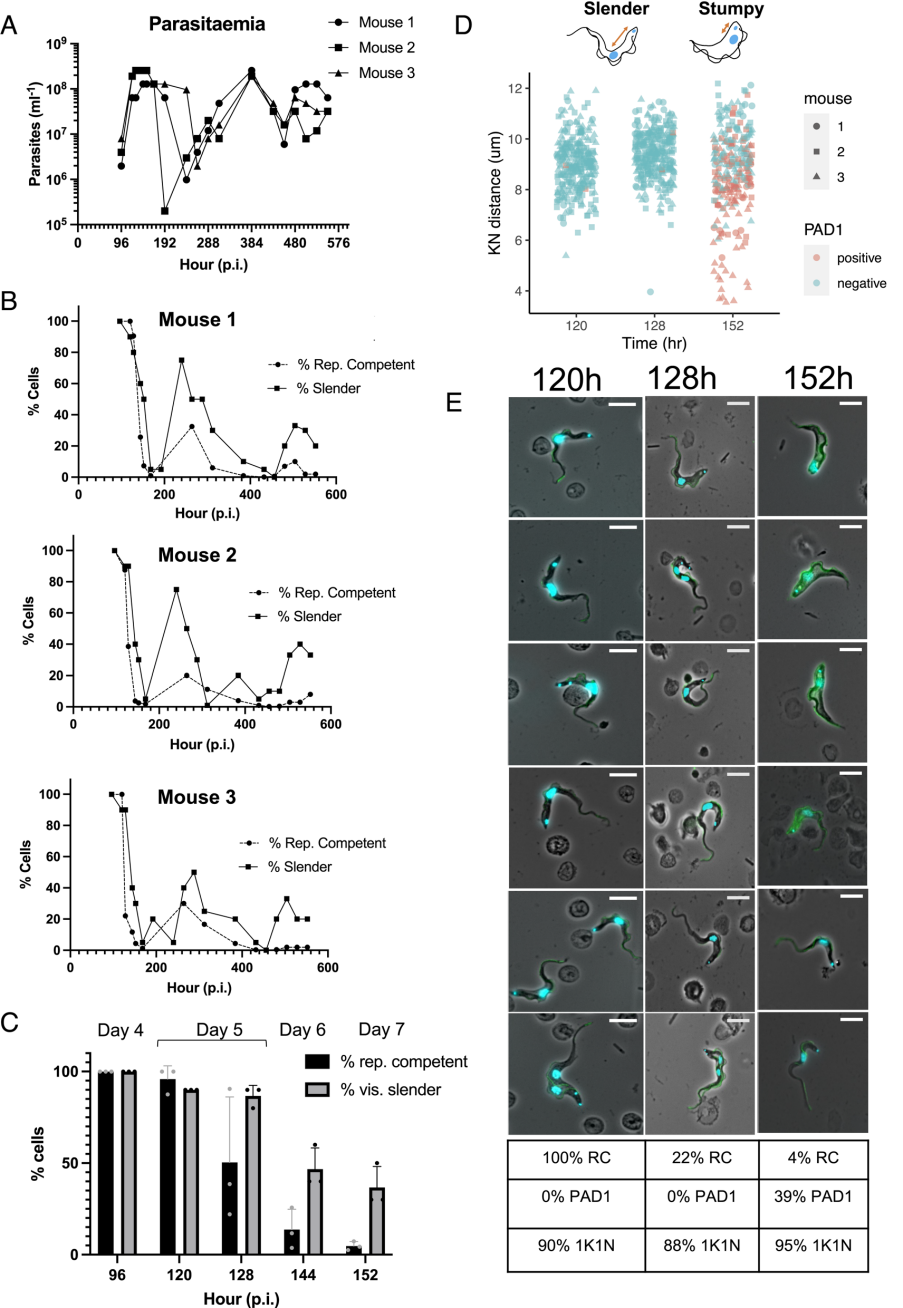
Nonproliferative forms dominate mouse infections. (*A*) Parasitemia of all mouse infections. (*B*) % replication (rep.) competent cells and morphology at different time points from a wild-type *T. brucei* Antat 1.1 90:13 infection of each mouse. (*C*) Comparison of the frequency of replication-competent cells and those with slender morphology p.i. over the first wave of parasitemia (n = 3). (*D*) Analysis of the kinetoplast-nucleus dimension (schematically represented for slender and stumpy forms above the plot) at 120 h, 128 h, and 152 h p.i.; PAD+ cells are in pink. Parasites from 120 h and 128 h are quantitated for all mice; for 152 h, when the populations comprised numerous stumpy cells, scoring focused on mouse 3 as a representative, with confirmatory analysis on a smaller number of parasites from mouse 1 and 2. (*E*) Representative phase contrast images of parasites at 120 h, 128 h, and 152 h of infection, counterstained for nucleus and kinetoplast (blue) and PAD1 (green). All cells are morphologically slender and PAD1 -ve at 120 h and 128 h. PAD1+ve stumpy and PAD1- slender cells are present at 152 h. Individual panels are shown in *SI Appendix*, Fig. S1 *A*–*C*. The Table shows the % RC, PAD1 +ve and cells with 1 kinetoplast and 1 nucleus (1K1N) for the respective infections. (Scale bar, 10 µm.)

To explore the commitment of trypanosomes to developmental arrest in the bloodstream we used an ex vivo plating assay, whereby pleiomorphic *T. brucei* AnTat 90:13 parasites from infections were carefully counted, serially diluted, and then, the proportion of parasites able to proliferate determined by their outgrowth in vitro. This established their “replicative competence”, i.e., the percentage of proliferating cells, or cells able to resume proliferation in vitro, at different stages of an infection in mice over 24 d (Fig. 1 *A*–*C*). Fig. 1 *A*–*C* demonstrate that early in the establishment phase of the infection [<120 h postinfection (p.i.)], there was an excellent correlation between the number of parasites in vivo and their replicative competence (100% ± 0%). This confirmed the ability of the assay to accurately quantitate replication-competent cells in the infection and demonstrated that the parasites were uncommitted to arrest at this point. Between 120 h and 128 h p.i., however, the proportion of replication-competent parasites declined rapidly to 13.7 ± 11.1% by 144 h (Fig. 1*C*), this coinciding with the peak of parasitemia in the first wave of infection (Fig. 1*A*). Following this, between 192 and 288 h, the parasites recrudesced, and 27.5 ± 6.6% were replication competent (RC). Beyond this point (384 h to 576 h), however, the parasitemia fluctuated between 1 × 10^7^ and 10^8^, but the proportion of replication-competent cells never exceeded 5.0 ± 4.3% (Fig. 1 *A* and *B*).

Replicative competence describes the proportion of parasites able to proliferate, whether derived from replicative, morphologically slender cells or from nonreplicative, morphologically slender or stumpy forms, that are not irreversibly committed to arrest and which can resume growth in vitro. Therefore, at each time point during infection, we related the morphology of the parasites to their replicative competence to assess the relationship between cell morphology and the commitment to arrest. Fig. 1 *B* and *C* demonstrate that until 120 h, the population was overwhelmingly slender in morphology, matching their replicative competence. However, between 128 and 152 h, morphologically slender cells always exceeded cells with replicative competence, demonstrating that the parasites had committed to arrest prior to their morphological transformation. These changes were very rapid such that parasites in one mouse (mouse 3) dropped from being 100 to 22% RC between 120 and 128 h p.i. (Fig. 1 *B* and *E*). Beyond 152 h, when stumpy cells were prevalent, the proportion of morphologically slender cells varied between 50% and 1%. Nonetheless, even when up to 30% of visually slender cells were detected late in the chronic phase of infection, only 2.5% of parasites exhibited replicative competence (Fig. 1*B*). For a more detailed assessment of morphology and developmental change, we investigated the expression of the stumpy marker protein PAD1 (protein associated with differentiation 1) (20), and the kinetoplast to nucleus (KN) distance (which is greater in slender forms than stumpy forms; shown schematically in Fig. 1*D*), focusing on the 120 h and 128 h time points when the rapid change in replicative competence occurred. Fig. 1 *D* and *E* show that there was no difference in either measure, proportion of cells with 1 kinetoplast and 1 nucleus (1K1N), or overall cell morphology, at these time points, despite the fall in replicative competence, contrasting with 152 h, when there was a clear change in both PAD1 expression and KN distance. Further images of fields of cells at 120 h, 128 h, 152 h, and 456 h are available in *SI Appendix*, Fig. S1 *A*–*D*.

In combination, these assays demonstrated, first, that the commitment to replicative arrest precedes PAD1 expression and morphological development to stumpy forms, second, that stumpy forms cannot resume proliferation once removed from the density-sensing signal in the blood, and, third, that after the early phase of the infection, replication-competent parasites in the bloodstream are scarce as a proportion of the population.

### Disrupting the Quorum-Sensing Pathway Increases Replicative Competence.

**Fig. 2.**
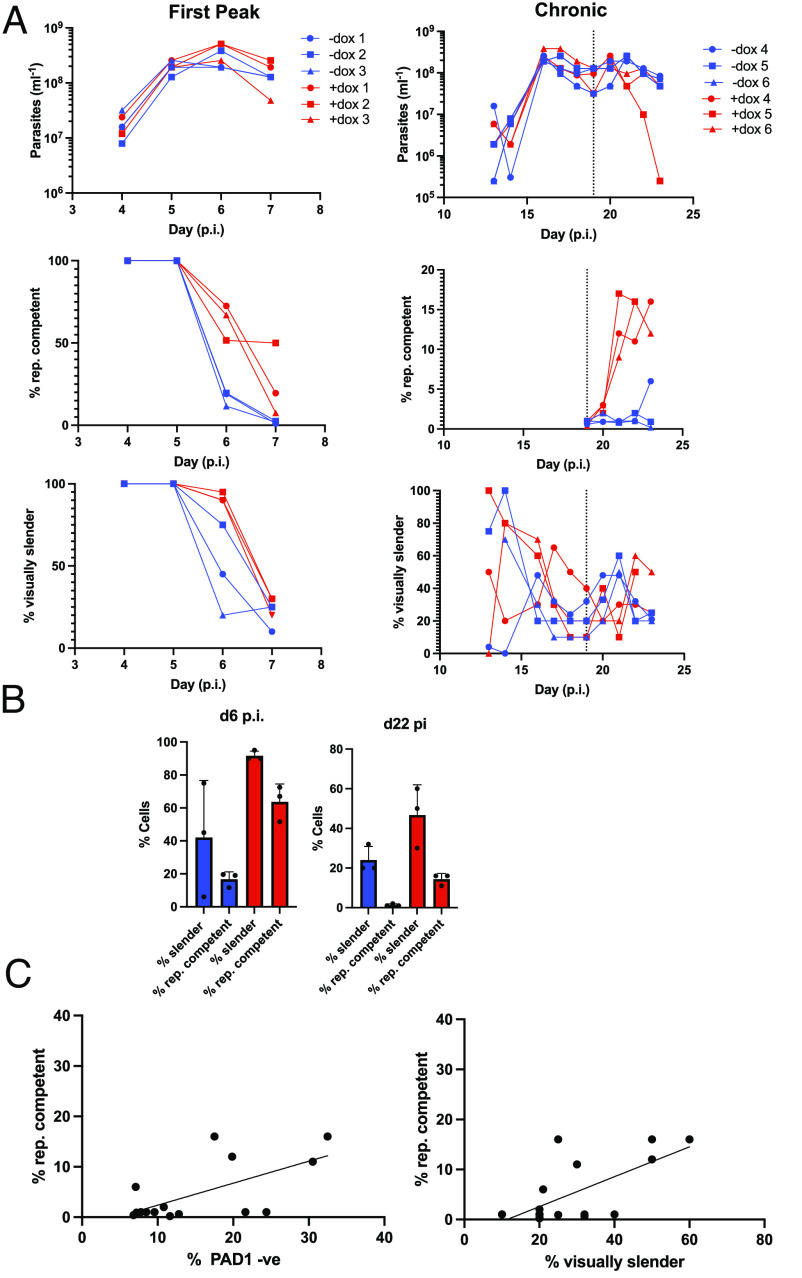
RNAi against HYP2 delays commitment to stumpy formation. (*A*) Parasitemia (*Top*), % replication-competent (*Middle*), and visually slender (*Bottom*) cells in the first peak (*Left*) or chronic phase (*Right*) of infection with (+dox; red) or without (−dox; blue) induction of RNAi against HYP2. The dashed line indicates the point that induction was started with doxycycline in chronic infections. (*B*) Percentage of cells with characteristics plotted in *A*, isolated after d6 p.i. or d22 p.i. to highlight the treatment differences between −dox (blue) and +dox (red). (*C*) Comparison between the proportion of replication-competent and PAD1 −ve (*Left*; *P* < 0.05) or visually slender (*Right*; *P* = 0.09) cells.

An alternative explanation for the loss of replicative competence during infections was a failure to grow in media after adaptation to the host environment. To eliminate this possibility, we exploited a genetic perturbation that reduces the quorum-sensing capacity of the parasites and so delays the development from slender to stumpy forms. We predicted that this would increase replicative competence of parasites later in infection if adaptation to the host was not an important factor for their growth ex vivo. Therefore, we initiated infections with a parasite line engineered for doxycycline-regulated RNAi-mediated silencing of HYP2, an identified component of the trypanosome quorum-sensing signaling pathway (21, 22). In the first peak (Fig. 2 *A*, *Left*), noninduced cells behaved as wild-type controls, with a reduction in replicative competence preceding the morphological change of the parasites. With HYP2 RNAi silencing, in contrast, the loss of replicative competence was reduced (F = 5.77, *P* = 0.003) as was the morphological transition (F = 6.5, *P* = 0.007), consistent with reduced quorum-sensing-mediated differentiation (Fig. 2 *A* and *B*).

The consequences of HYP2 RNAi were further evaluated in the chronic phase of the infections, between d19 and d23, when replicative competence is very low. (Fig. 2 *A*, *Right*). Although there was variation between mice, by d22 p.i., there was an increase in both morphologically slender cells and those exhibiting replicative competence (noninduced 1.33% ± 0.57; induced 14.33% ± 2.88; F_4,18_ = 12.81, *P* < 0.0001; Fig. 2 *A*, *Right* and Fig. 2*B*). Extending the analysis beyond morphology for both induced and uninduced parasites further demonstrated that PAD1 expression (F = 15.48, *P* = 0.002) and slender morphology (F = 3.31, *P* = 0.09) showed an inverse correlation with replicative competence but that the relationship was not 1:1; many PAD1 negative, morphologically slender cells were not RC (Fig. 2*C*).

Thus, more slender form parasites derived from acute and chronic infections can successfully proliferate in vitro if quorum sensing is reduced, eliminating host adaptation as an explanation for the low replicative capacity in wild-type cells. Instead, regardless of their morphology, after initial establishment, parasites show extremely limited replicative competence due to their commitment to development to stumpy forms.

### The Stumpy-Associated Gene Expression Program is Activated before Irreversible Commitment.

To investigate the characteristics of cell types (replicative slender, arrested stumpy, and uncommitted or committed cells of any morphology) in acute and chronic stages of infection, we exploited single-cell RNA sequencing with the Chromium platform (18). Parasites were isolated from day 7 and day 23 of infection, with HYP2 RNAi being induced or not several days prior to harvesting (i.e., at day 0 in acute infection and day 19 for the chronic infections). After quality control filtering 3,826 transcriptomes remained for d7-dox, 7,721 for d7+dox, 8,435 for d23-dox, and 7,721 for d23+dox. All samples were integrated prior to dimension reduction and clustering analysis (*SI Appendix*, Fig. S2).

**Fig. 3.**
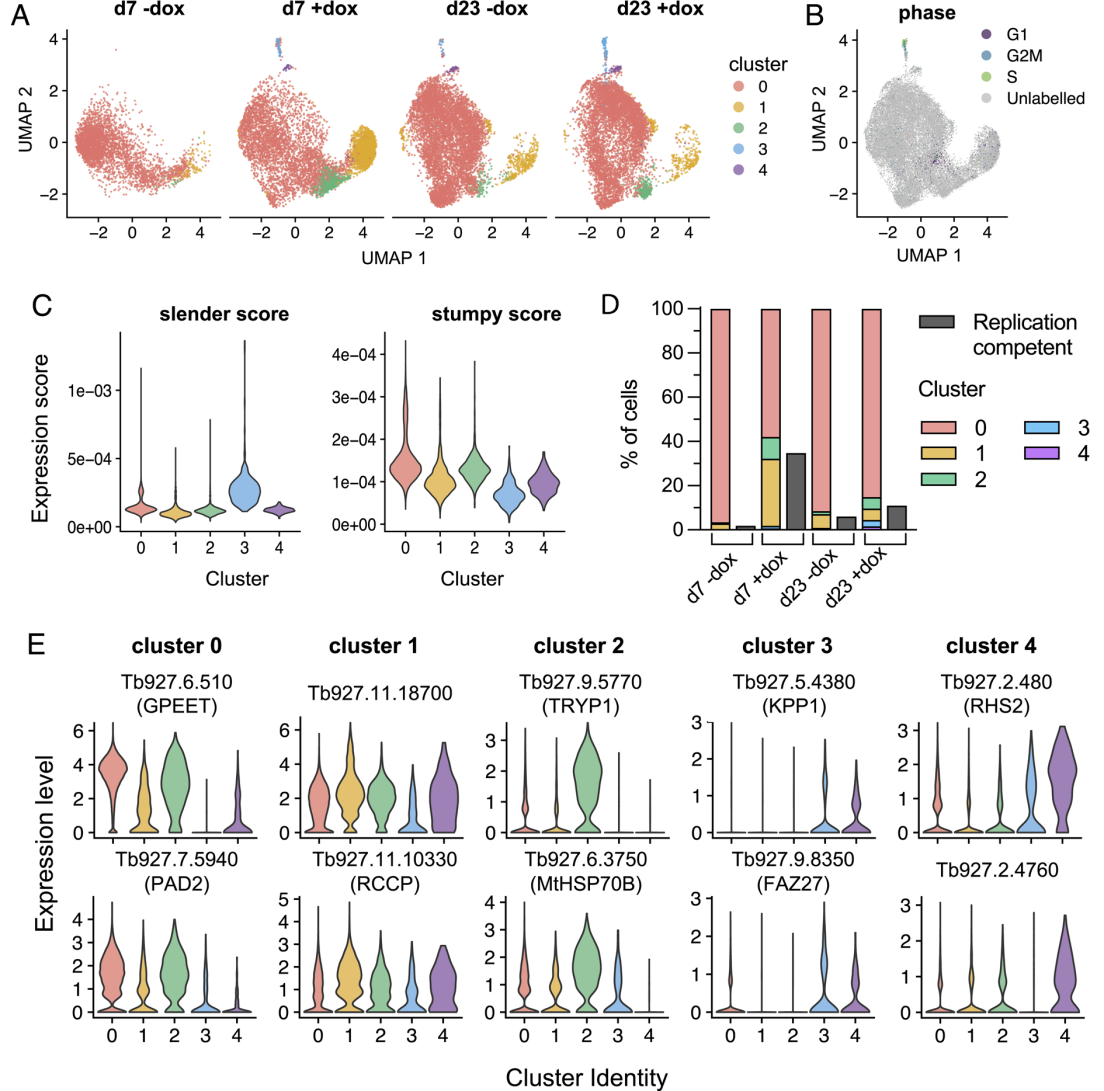
Single-cell RNA-seq reveals the scarcity of parasites with a slender-associated transcriptome. (*A*) UMAP plots of cell transcriptomes at day 7 and day 23 p.i., ±dox induction of HYP2 RNAi. Each point is one cell, colored by cluster identity. Key to the right is applicable to each plot. (*B*) Cells labeled by cycle phase, for all samples. (*C*) The “slender score” and “stumpy score” consisting of the average expression of associated marker genes for each cluster identified in panel *A*, for all samples. (*D*) Comparison of the proportion of each cluster with the % of replication-competent cells in each sample. (*E*) Expression of two cluster-enriched marker genes for clusters 0 to 5, analyzed with respect to each cluster. Markers highlighted are Tb927.6.510 (GPEET) (25); Tb927.7.5940 (PAD2) (20); Tb927.11.18700 (Hypothetical protein, conserved); Tb927.11.10330 (regulator of chromosome condensation 1-like protein) (26); Tb927.9.5770 (tryparedoxin peroxidase) (27); Tb927.6.3750 (mitochondrial HSP70B) (28); Tb927.5.4380 (Kinetoplastid-specific Protein Phosphatase 1) (29); Tb927.9.8350 (FAZ27) (30); Tb927.2.480 (Retrotransposon hotspot protein 2); and Tb927.2.4760 (Hypothetical protein, conserved).

Across all four samples, we identified 5 clusters of cells defined with distinct marker genes (Fig. 3*A*). To broadly characterize the clusters, we used expression levels of a panel of existing marker genes (18) to assign “slender” and “stumpy” scores to each cell, and also, the respective clusters were labeled according to cell cycle marker gene expression (Fig. 3*B* and *SI Appendix*, Fig. S3). As expected from the numbers of replication-competent cells at both d7 and d23, only a small minority of parasites showed high expression of slender marker genes which nearly exclusively belonged to cluster 3 (Fig. 3*C*). This cluster, which we consider “true” slender cells, comprised <2.7% of cells in any sample and was the only cluster exhibiting expression of markers consistent with active cell cycle progression (Fig. 3*C* and *SI Appendix*, Fig. S3). All the other clusters showed low slender scores and higher expression of stumpy marker genes, although cluster 4 and cluster 1 parasites showed notably lower stumpy scores than cluster 2 and cluster 0. Cluster 0, the most common group of cells in all samples, is associated with high stumpy scores and represents terminally committed stumpy forms. Consistent with this, these cells were marked by relatively high expression, for example, of EP and GPEET procyclins, ZC3H20, PAD2, and ZFPs, each of which has been found to be up-regulated as parasites prepare for transmission or undergo stumpy formation (10, 23). The cyclin F box2 protein was also elevated, this being associated with VSG mRNA stability (24).

Importantly, the proportion of cells in each sample that was RC after ex vivo plating was always higher than the proportion of true slender cells (cluster 3), implicating the presence of cells in other clusters that were not yet irreversibly committed to arrest (Fig. 3*D*). By examining the cumulative representation of distinct clusters in each experimental condition (acute, chronic, HYP2 induced or not), we found that a composite score, composed of a combination of cells belonging to clusters 1, 3, and 4, provided an excellent match to the corresponding proportion of cells that were RC in each sample (Fig. 3*D*). Thus, their number increased with HYP2 RNAi and was lower when HYP2 was not silenced, supporting their identification as uncommitted cells. Critically, despite the anticipation that they are RC, the transcriptomic profiles of cells belonging to clusters 1 and 4 do not match those of cluster 3, the true slender cells, suggesting that they have embarked on the developmental program to stumpy forms. Interestingly, although cluster 1 is similar to the transcriptome profile of stumpy forms (cluster 0), it exhibited higher expression of Tb927.11.18700, a reported target of the Grumpy snoRNA implicated in stumpy development (31), a chromatin remodeling ISWI complex component (32), and a telomere-associated protein (33) linked to expression site silencing, a feature of development to stumpy forms (34). Notably, no cell cycle markers were elevated in clusters 1, 2, and 4 (Fig. 3*B* and *SI Appendix*, Fig. S3) demonstrating an absence of their replicative activity in the bloodstream, despite the ability of these uncommitted cells to resume growth when isolated and cultured ex vivo. Comparative datasets for each cluster with respect to the overall population are available for interrogation in Dataset S1, whereas two enriched marker transcripts for each cluster are shown in Fig. 3*E*. A comparison between the composition of each cluster either early or in chronic infections is shown in Dataset S2.

**Fig. 4.**
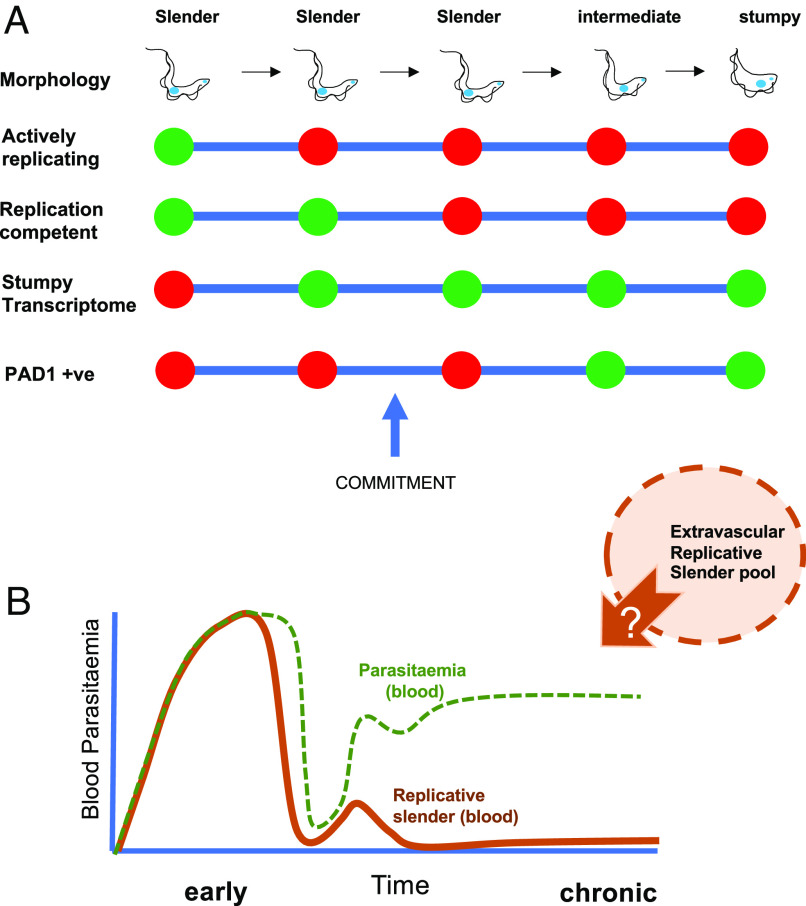
The developmental commitment of slender forms and their contribution to the infection dynamic. (*A*) Events in the development of slender forms toward stumpy forms, highlighting that the acquisition of a stumpy-like transcriptome and irreversible commitment precedes morphological change and PAD1 protein expression. (*B*) Model for the source of blood parasites given the scarcity of replication-competent slender forms in the vasculature.

In combination, these data quantitatively reveal that there is an exceptionally small population of true slender cells in active replication in the blood, together with a population of uncommitted cells that do not proliferate, share the expression of many genes expressed in stumpy forms, and yet are capable of reentering the cell cycle if removed from the bloodstream environment (Fig. 4). Finally, the remaining and dominant proportion in the bloodstream are terminally arrested stumpy forms.

## Discussion

The established view of trypanosomes in the mammalian bloodstream is that slender forms proliferate to sustain the infection and generate antigenic diversity while stumpy forms are irreversibly arrested and preadapted for transmission for tsetse flies. The direct evidence for both of these elements of the infection dynamic, however, is limited. In this study, we found that morphologically stumpy forms were incapable of reentering proliferation if removed from the quorum-sensing signal. Moreover, by careful temporal analysis during the first wave of parasitemia, we could establish that the commitment to arrest precedes morphological transformation such that cells indistinguishable from proliferative bloodstream slender forms early in the parasitemia lost replicative competence significantly before morphologically stumpy forms appeared. By day 7, single-cell analysis showed not one of 3,826 cells (d7-dox) had a transcriptome representative of true slender (cluster 3) cells (18), and cells with replicative competence were generally present at less than 2% of the population. Analysis in the chronic phase of infection confirmed that stumpy forms adapted for transmission dominated, as previously proposed based on population-level analysis (19), but importantly revealed at the level of individual cells that slender forms capable of proliferation were exceptionally rare, representing only 0.33% of the bloodstream trypanosome population at d22. Moreover, by single-cell RNA sequence analysis, only around two-thirds of even this small proportion appeared to be undergoing active proliferation. Overall, we can conclude that slender cells undergo irreversible commitment to stumpy formation before any morphological change (Fig. 4*A*) and that the proliferation of slender forms in the bloodstream makes a much smaller than anticipated contribution to the maintenance of infection (Fig. 4*B*). Indeed, the proportion of replicative slender forms is even less at the individual cell level than previously estimated based on PAD1 expression and mathematical modeling (19).

Previous analyses of the irreversibility of stumpy cell cycle arrest are based on in vitro studies (14, 15), mathematical modeling of the parasitemia (19, 35), and the inference that stumpy enriched populations are quantitatively less infective to naïve animals (17). In our assays, the ability to proliferate ex vivo was analyzed, and the parasites were found to precipitously lose replicative competence from approximately 120 h of infection in mice, whereafter it remained very low. We considered that this could be contributed to by the parasites becoming adapted to the mammalian host environment and so less able to efficiently proliferate ex vivo. This possibility, however, was eliminated by silencing a component of the quorum-sensing pathway; under these conditions, the capacity of the cells to become stumpy was reduced in vivo, and, correspondingly, the replicative competence of the parasites increased. Importantly, this was the case not only early in infection but also 20 d after growth of the parasites in vivo. Thus, the parasites develop to stumpy forms during the course of the parasitemia, and once generated, these cells cannot reenter proliferation and are terminally committed to cell cycle arrest, at least until development to the next life cycle stage in the tsetse fly. Definitively, therefore, stumpy formation is irreversible.

From quantitative analysis of replication competence and the transcriptome profile of the parasite population, replicative slender cells frequently represent less than 1% of trypanosomes in the chronic phase of infection, which is more typical of natural long-term infections. Is this sufficient for bloodstream parasites to sustain the infection? Although many parameters are uncertain, our data suggest not. For example, between d19 and d21, the proportion of replication-competent cells never exceeded 1%, which could not generate the two-fold increase in total parasitemia observed during this chronic period, particularly when combined with an expected decay in the stumpy forms through senescence (12). Although short transient bursts of replication could be missed in our temporal analysis, and the lower parasitemias of livestock infections could facilitate a higher prevalence of replicative cells, in mice replicative slender cells appear too scarce. One explanation to sustain the infection is that the population is replenished from niches elsewhere. Characterized extravascular locations for trypanosomes include the skin, lungs, and adipose tissue (36–38), and the migration between the adipose tissues and blood has recently been estimated to be 11% of blood parasites/per milligram of tissue/day, highlighting that there is significant, though restricted, potential for exchange (39). These extravascular compartments have recently been identified as sites of elevated antigenic diversity (40) and, if this is combined with enhanced replicative capacity, could seed parasites into the bloodstream which have already initiated development to stumpy forms. With high parasitemias, these would rapidly arrest and transition to stumpy forms in response to the existing QS signal in the blood, but with low parasitemias, there is the potential for a reversion to proliferation for any cells that are not yet irreversibly committed. The site of commitment to stumpy formation (tissue, blood) could therefore vary depending on the nature of the infection but, based on our experiments, creates a model where the parasites disseminated in the tissues serve to populate the circulation with a pool of parasites optimized to transmit to hematophagous tsetse flies.

Single-cell RNA sequencing allowed the molecular characteristics of parasites in the bloodstream population to be interrogated in the early and chronic phase of infection. Moreover, inclusion in the analysis of cells after HYP2 silencing informed on the relationship between the transcriptome profile of the respective cell clusters and their replicative potential. Specifically, beyond the majority of cells with a stumpy transcriptome (cluster 0) and the tiny proportion of true slender cells (cluster 3), there was a significant group of cells (clusters 1 and 2) that were similar to, but distinct from, stumpy forms plus a small group of cells similar to replicative slender forms (cluster 4). Consistently, the combined proportion of these clusters was equivalent to the proportion of cells which retained proliferative competence ex vivo (with their proportion increasing as quorum sensing is reduced by HYP2 silencing). We interpret these clusters as representing cells which are not committed to irreversible arrest and yet have undergone significant developmental adaptation, which is reversible including at the level of the transcriptome. Although able to proliferate ex vivo if removed from the quorum-sensing signal, these parasites would remain arrested in vivo and progress to full commitment and stumpy formation without regaining replicative capacity—unless removed from the signal, for example, by (re)invading a low-density extravascular niche. This generates a hierarchy of development whereby morphologically slender forms are initially proliferative, then reversibly activate a transcriptome profile similar to stumpy forms, followed by irreversible commitment to arrest and finally morphological transition to the stumpy form (Fig. 4*A*).

Our data address several key aspects of the infection biology of trypanosomes in the mammalian blood. First, they formally demonstrate that the development of trypanosomes to stumpy forms entails terminal developmental arrest in the mammalian host. Second, by temporal analysis of replicative competence through the first wave of parasitemia and single-cell transcriptome analysis, the commitment to arrest and development was found to precede PAD1 expression and morphological change to stumpy forms. Third, our finding that replicative slender forms in the bloodstream are exceptionally rare challenges the ability of the bloodstream parasite population to maintain the infection or drive antigenic variation, complementing the description of elevated antigenic diversity in extravascular niches (40). Furthermore, although it has been reported that slender, PAD1-negative, cells have the potential for onward development in flies (41), our data suggest that many such cells would in fact be already committed to stumpy development regardless of their morphology and any replicative slender cells would be insufficiently prevalent to contribute meaningfully to transmission. Finally, by the single-cell comparison of parasite populations early and late in the infection (Dataset S2), we provide a platform for the future biological analysis of specific characteristics of parasites in the chronic stage of infection. These have been almost entirely neglected to date but are most relevant for infections in the field.

## Materials and Methods

### Mouse Infections.

In vivo experiments were performed under UK Home Office license number (PP2251183) approved after review at the University of Edinburgh ethical review committee.

For all experiments, we used 10 to 12-wk-old female Balb/c mice (Charles River). For wild-type infections, mice were infected with 100 cells of pleiomorphic *T.brucei* EATRO1125 AnTat 1.1 90:13 (42) (Table 1). For the HYP2 RNAi infections, mice were infected with 100 cells of a previously described *T. brucei* EATRO1125 AnTat1.1 90:13 cell line with a doxycycline-inducible RNAi targeting *Tb*HYP2 (21). In both cases, frozen stocks were cultured for 2 d prior to infection to ensure that all cells in the inoculum were viable. Two experiments were carried out using the inducible HYP2 RNAi line: In the first experiment, induction of RNAi commenced on d0 of infection and was terminated at d7 p.i. In the second, RNAi against HYP2 was induced on d19 of infection and was terminated on d23. In each case, induction was achieved by addition of doxycycline to drinking water (200 μg/mL in 5% sucrose). Noninduced controls received drinking water with 5% sucrose. In all mouse infections, mice were blood sampled regularly following d3 p.i. by tail snip. Parasitemia was assessed using Herbert and Lumsden matching charts for fields of view at 40× magnification (43). Morphology was also scored: The number of clearly slender parasites was counted for at least three fields of view and used to create a % of slender cells for each day. Slender assignment was scored based on the morphology, the length of flagella, and the motility of cells and was always done within 30 min of blood sampling. All blood sampling was kept within UK Home Office limits as specified in the relevant project license. In addition to rapid scoring on days for analysis of replication competence, we also kept around 5 µL of blood in 500 µL of HMI-9 media. This was kept warm for subsequent use in plating assays and for making blood smears.

**Table 1.** Key Resource Table with source and catalogue identifiers for all experiments

Reagent or resource	Source	Identifier
Antibodies		
Goat Anti-Rabbit IgG (H+L) Antibody, Alexa Fluor 488 Conjugated	Invitrogen	CAT#A-1108, RRID:AB_143165
Anti-PAD1	Dean et al., (20)	N/A
Critical commercial assays		
Chromium Single Cell 3′ v3.1	10× Genomics	Guide CG000204
Deposited data		
Single-cell transcriptomic raw fastq data	This paper, EBI-ENA	Project PRJEB60851
Single-cell transcriptomic cell × counts matrices	This paper, Zenodo	DOI: 10.5281/zenodo.7778583
Experimental models: Cell lines		
*Trypanosoma brucei* EATRO 1125 AnTat1.1 90:13	Engstler and Boshart (42)	N/A
*T. brucei* EATRO 1125 AnTat1.1 90:13_RNAi_HYP2	Mony et al. (21)	Gene ID: Tb927.9.4080
Experimental models: Organisms/strains		
*Mus musculus*: strain balb/c, female	Charles River	RRID: IMSR_APB:4790
Software and algorithms		
GraphPad Prism	www.graphpad.com	RRID: SCR_002798
R	https://www.r-project.org/	RRID: SCR_001905
RStudio	https://rstudio.com/	RRID: SCR_000432
Cellranger version 7	10× Genomics	N/A
Seurat version 4.1.0	Hao et al. (44)	RRID: SCR_007322
Complete scRNA-seq analysis code	This paper, Zenodo	DOI: 10.5281/zenodo.7778583
Other		
TritrypDB database	http://tritrypdb.org/tritrypdb/	N/A
10× Genomics chromium plus genetic analyzer	10× Genomics	SCR_019326
Anion exchange cellulose (DE52)	Whatman	Cat# 4057200
HMI-9 Medium	Life Technologies	Cat#074-90915

### Plating Assay.

We developed a plating assay for assessment of ex vivo proliferation of parasites from mice. Following blood sampling and parasitemia scoring, we used the diluted blood sample in media to fill plates with known numbers of cells. We used an improved Neubauer hemocytometer to accurately count the parasites in each 500 µL. The counts were used to create a dilution for a final stock with a fixed concentration of 1 cell/µL in 5 mL total volume. Since parasitemia is commonly around 1 × 10^7^ cells per ml in blood, this rapid dilution to 1,000 per ml represents a 10,000-fold dilution, effectively removing the density-dependent oligopeptide differentiation signal. The stock was then used to fill a round bottom 96-well plate with the following cell numbers: 50/well, 20/well, 5/well, 2/well, and 1/well. The wells were filled with HMI-9 media to 100 µL total volume. In the HYP2 RNAi experiment, we continued doxycycline treatment from mice into the 96-well plate. After seeding, the plates were left untouched for 5 to 7 d; then, each well was assessed for an outgrowing culture of parasites in a binary fashion. After 5 to 7 d, there was almost always nothing or a viable culture in each well. On rare occasions where only a few cells were in a well, it was left a further 3 d and then rescored. Wells around the edges of the plate were not used as pilot work indicated that these grew less successfully. Thus, there were 12 replicates for each seeding. The number of wells was counted for each seeding that developed outgrowth, and the percentage growing for each condition was calculated. Since only one competent cell is required for outgrowth, the probability (P) theory was used to calculate the estimated proportion of replication-competent cells using the following formula: P(at least one cell replicative) = 1 − P(failure in one trial)^n^, where n is the total number of trials. We used an average of the values of replication-competent cells across the seedings where there was variation in outgrowth, avoiding those where everything or nothing grew: these seedings do not offer enough variation to create an accurate estimate.

### Microscopy.

In addition to rapid scoring of “slenderness” for a subset of samples, leftover cells from the stock used to seed the plating assay were used to examine the types of cells present in the samples in detail with immunofluorescent microscopy. These were 120 h and 128 h p.i. in the WT experiment and days 5, 7, 19, 22, and 23 in our HYP2 RNAi experiment. On each of these days, after quenching any mouse antibodies for at least 30 min in HMI-9, the blood was centrifuged (10 min at 400 g), and the supernatant was removed. The remaining blood and parasite pellet was used to make 2 blood smears per mouse. Smears were air dried before being fixed and stored in methanol at −20 °C. Cell cycle analysis and PAD1 protein expression analysis were carried out by staining ice-cold methanol fixed cells with DAPI (100 ng/mL) and an anti-PAD1 antibody (20) as previously described (22). Images were made of all slides at 63x magnification, aiming for at least 100 cells for each slide. The cell cycle status was determined by counting the number of kinetoplasts and nuclei in each cell (KN score) and scoring for the presence/absence of staining with the PAD1 antibody. Positive cells were classed based on any PAD1 staining that was not restricted to only the flagella or flagellar pocket. For a nonbinary description of differentiation, the KN distance was also measured from all images of cells in 1K1N, providing a quantitative proxy for the change from slender to stumpy as the cell reconfigures morphologically. The KN distance measuring focused on the crucial 120- and 128-h time points highlighted in the WT AnTat 90:13 mouse experiments, when the cells appeared to be morphologically identical and slender. A smaller number of cells (>60 in all cases) from the same mice were analyzed at 152 h p.i. as a control: At this time point, cell morphology was clearly mixed in each mouse, with some clearly transitioned to full stumpy morphology. Using ImageJ (45), a script was used to automatically measure the distance between the centers of two particles in the DAPI-stained images. The distance in pixels was converted to µm by dividing by 6.106155.

### Statistical Analyses.

For the mouse experiments, three animals per treatment were used. With effects sizes similar to those previously observed for RNAi mediated loss of developmental competence (0.637 to 1.804; e.g., ref. (21)), a sample size of three per treatment provided sufficient power to test for treatment differences. To test for differences in replicative competence mediated by treatment, a one-way repeated measures ANOVA was constructed with interest in the time* treatment (+ or – dox) interaction. To assess how differences in characteristics at the population level (%2K1N, 2K2N*P; % PAD1 +ve*; % visually slender) were related to the percentage of replication-competent cells for a given time point, a multiple linear regression analysis was constructed. This model assessed the significance of relationships between each variable, while holding treatment groups as fixed factors. The models were simplified by removing nonsignificant terms until only interactions with a *P* value of < 0.1 remained. In all cases, the simplified models provided a better fit to the observed data than larger models, as assessed by sum of squares (smaller is better). Blinding was not carried out. *P* values of less than 0.05 were considered statistically significant in all cases. For scRNA-seq differential expression tests, a significance threshold of adjusted *P*-value < 0.05 was used.

### Chromium (10× Genomics) Library Preparation and Illumina Sequencing.

Parasites were purified from terminal blood samples using diethylaminoethyl-cellulose columns (46). Following isolation from host cells into PSG buffer (1× PBS + 1% D-glucose, pH 7.8), centrifugation at 400 × g for 10 min was used to pellet parasites which were resuspended in 1 mL HMI-9 supplemented with 20% fetal calf serum (FCS). Parasites were counted with a hemocytometer and adjusted to ~2 × 10^6^ cells/mL. Parasites were then mixed 1:1 with 2× freezing media (HMI-9 with 20% glycerol) and frozen at −80 °C wrapped in cotton wool to slow freezing. After 24 to 72 h, parasites were moved to liquid nitrogen storage. For Chromium scRNA-seq (single cell RNA sequencing) (10× Genomics), samples were transported on dry ice before slow defrosting to limit cell death using the previously validated protocol (47). For each sample, parasites were combined from 2 to 3 mice in equal proportion depending on sample viability, only samples with >90% live, motile, parasites were used for sequencing. Samples day 7 -dox (n = 3) and +dox (n = 2) were processed in one batch. The first run of day 23 -dox (n = 3) and day 23 +dox (n = 2) samples resulted in low cell recovery; 1,084 and 623 cells, respectively. Therefore, a second batch of remaining samples (n = 1 for -dox and n = 2 for +dox) were processed for day 23, and two batches were combined for day 23 samples. Sample preparation was performed with the 3′ Gene Expression kit (version 3.1 chemistry), and sequencing was performed with Illumina NextSeq 2000, to generate 28 bp ×4 130 bp paired reads to high depth: 62,083 mean reads per cell for day 7 −dox, 61,345 mean reads per cell for day 7 +dox, 81,559 mean reads per cell for day 23 −dox, and 91,441 mean reads per cell for day 23 +dox. All fastq files are available on ENA (European Nucleotide Archive) under project PRJEB60851.

### scRNA-seq Data Processing and Analysis.

Data were mapped to the *T. brucei* TREU927 with extended UTR sequences (18) using Cellranger software version 7 to generate counts matrices. As the day 7 -dox sample showed a higher than expected number of droplets with low total RNA, we controlled for potential free RNA in the samples with SoupX (48). Resulting counts were further quality control filtered to remove low-quality transcriptomes and likely multiplets (*SI Appendix*, Fig. S2). For each sample, variable genes were selected before data were log2 normalized and scaled using previously described methods (18). All four samples were integrated using Seurat version 4 (44) before dimension reduction and clustering analysis were performed. Differential expression testing between clusters was performed using MAST (Model-based Analysis of Single-cell Transcriptomics) (49). Average expression scores were calculated using the AddModuleScore function from Seurat which finds the average expression levels of marker gene set across each cell, subtracted by the aggregated expression of a randomly selected control gene set (50). For cell cycle phase scores, previously published marker genes were used, and early and late G1 markers were combined (51). To generate slender and stumpy marker gene sets, the single-cell transcriptomes of in vitro generated slender and stumpy forms (18) were compared to find genes with the highest fold change (>2) with the MAST differential expression test, resulting in 414 slender and 110 stumpy marker genes. All code and counts matrices are available on Zenodo (10.5281/zenodo.7778583) (52).

## Supplementary Material

Appendix 01 (PDF)

Dataset S01 (XLSX)

Dataset S02 (XLSX)

## Data Availability

For scRNAseq data, the raw fastq is registered with the ENA (European Nucleotide Archive) (https://www.ebi.ac.uk/ena/browser/home) under the study reference PRJEB60851 (https://www.ebi.ac.uk/ena/browser/view/PRJEB60851) (53). The original code is available at https://tinyurl.com/28ydamyy (52).

## References

[1] F. Giordani, L. J. Morrison, T. G. Rowan, H. P. De Koning, M. P. Barrett, The animal trypanosomiases and their chemotherapy: A review. Parasitology **143**, 1862–1889 (2016). 27719692 10.1017/S0031182016001268 PMC5142301

[2] B. Rotureau, J. Van Den Abbeele, Through the dark continent: African trypanosome development in the tsetse fly. Front. Cell Infect. Microbiol. **3**, 53 (2013). 24066283 10.3389/fcimb.2013.00053 PMC3776139

[3] D. Bruce, D. Harvey, A. Hamerton, J. Davey, L. Bruce, The morphology of the Trypanosome causing disease in man in Nyasaland. Proc. R. Soc. B. **85**, 423–433 (1912).

[4] L. M. McLintock, C. M. Turner, K. Vickerman, Comparison of the effects of immune killing mechanisms on *Trypanosoma brucei* parasites of slender and stumpy morphology. Parasite Immunol. **15**, 475–480 (1993). 8233562 10.1111/j.1365-3024.1993.tb00633.x

[5] K. R. Matthews, Trypanosome signaling-quorum sensing. Annu. Rev. Microbiol. **75**, 495–514 (2021). 34348028 10.1146/annurev-micro-020321-115246

[6] M. Robertson, Notes on the polymorphism of *Trypanosoma gambiense* in the blood and its relation to the exogenous cycvle in Glossina palpalis. Proc. R Soc. B **85**, 241–539 (1912).

[7] F. Rojas, K. R. Matthews, Quorum sensing in African trypanosomes. Curr. Opin. Microbiol. **52**, 124–129 (2019). 31442903 10.1016/j.mib.2019.07.001

[8] M. R. Mugnier, The in vivo dynamics of antigenic variation in *Trypanosoma brucei*. Science **349**, 247–247 (2015). 25814582 10.1126/science.aaa4502 PMC4514441

[9] K. R. Matthews, S. Larcombe, Comment on “Unexpected plasticity in the life cycle of *Trypanosoma brucei*”. eLife **11**, e74985 (2021). 10.7554/eLife.74985 PMC8806180 35103595

[10] B. Szoor, E. Silvester, K. R. Matthews, A leap into the unknown–Early events in African trypanosome transmission. Trends Parasitol. **36**, 266–278 (2020). 32014419 10.1016/j.pt.2019.12.011

[11] C. M. Turner, N. Aslam, C. Dye, Replication, differentiation, growth and the virulence of *Trypanosoma brucei* infections. Parasitology **111**, 289–300 (1995). 7567097 10.1017/s0031182000081841

[12] C. E. Dewar , Mitochondrial DNA is critical for longevity and metabolism of transmission stage *Trypanosoma brucei*. PLoS Pathog. **14**, e1007195 (2018). 30020996 10.1371/journal.ppat.1007195 PMC6066258

[13] W. H. Lumsden, Infectivity of salivarian trypanosomes to the mammalian host. Acta Trop. **29**, 300–320 (1972). 4405440

[14] E. Vassella, B. Reuner, B. Yutzy, M. Boshart, Differentiation of African trypanosomes is controlled by a density sensing mechanism which signals cell cycle arrest via the cAMP pathway. J. Cell Sci. **110**, 2661–2671 (1997). 9427384 10.1242/jcs.110.21.2661

[15] B. Reuner, E. Vassella, B. Yutzy, M. Boshart, Cell density triggers slender to stumpy differentiation of *Trypanosoma brucei* bloodstream forms in culture. Mol. Biochem. Parasitol. **90**, 269–280 (1997). 9497048 10.1016/s0166-6851(97)00160-6

[16] B. Hamm, A. Schindler, D. Mecke, M. Duszenko, Differentiation of *Trypanosoma brucei* bloodstream trypomastigotes from long slender to short stumpy-like forms in axenic culture. Mol. Biochem. Parasitol. **40**, 13–22 (1990). 2348830 10.1016/0166-6851(90)90075-w

[17] M. P. Cunningham, K. van Hoeve, W. H. R. Lumsden, Variable infectivity of organisms of the T. brucei subgroup during acute relapsing infections in rats, related to parasitaemia, morphology and antibody response. Ann. Rep. EA Tryp. Res. Org. 21 (1963).

[18] E. M. Briggs, F. Rojas, R. McCulloch, K. R. Matthews, T. D. Otto, Single-cell transcriptomic analysis of bloodstream *Trypanosoma brucei* reconstructs cell cycle progression and developmental quorum sensing. Nat. Commun. **12**, 5268 (2021). 34489460 10.1038/s41467-021-25607-2 PMC8421343

[19] P. Macgregor, N. J. Savill, D. Hall, K. R. Matthews, Transmission stages dominate trypanosome within-host dynamics during chronic infections. Cell Host Microbe. **9**, 310–318 (2011). 21501830 10.1016/j.chom.2011.03.013 PMC3094754

[20] S. Dean, R. Marchetti, K. Kirk, K. R. Matthews, A surface transporter family conveys the trypanosome differentiation signal. Nature **459**, 213–217 (2009). 19444208 10.1038/nature07997 PMC2685892

[21] B. M. Mony , Genome-wide dissection of the quorum sensing signalling pathway in *Trypanosoma brucei*. Nature **505**, 681–685 (2014). 24336212 10.1038/nature12864 PMC3908871

[22] E. Silvester, J. Young, A. Ivens, K. R. Matthews, Interspecies quorum sensing in co-infections can manipulate trypanosome transmission potential. Nat. Microbiol. **2**, 1471–1479 (2017), 10.1038/s41564-017-0014-5. 28871083 PMC5660621

[23] K. R. McWilliam, A. Ivens, L. J. Morrison, M. R. Mugnier, K. R. Matthews, Developmental competence and antigen switch frequency can be uncoupled in *Trypanosoma brucei*. Proc. Natl. Acad. Sci. U.S.A. **116**, 22774–22782 (2019). 31636179 10.1073/pnas.1912711116 PMC6842576

[24] G. Bravo Ruiz, M. Tinti, M. Ridgway, D. Horn, Control of variant surface glycoprotein expression by CFB2 in *Trypanosoma brucei* and quantitative proteomic connections to translation and cytokinesis. mSphere **7**, e0006922 (2022). 35306877 10.1128/msphere.00069-22 PMC9044945

[25] P. Butikofer, E. Vassella, A. Mehlert, M. A. Ferguson, I. Roditi, Characterisation and cellular localisation of a GPEET procyclin precursor in *Trypanosoma brucei* insect forms. Mol. Biochem. Parasitol. **119**, 87–95 (2002). 11755189 10.1016/s0166-6851(01)00398-x

[26] T. Stanne , Identification of the ISWI chromatin remodeling complex of the early branching eukaryote *Trypanosoma brucei*. J. Biol. Chem. **290**, 26954–26967 (2015). 26378228 10.1074/jbc.M115.679019 PMC4646403

[27] E. Tetaud, A. H. Fairlamb, Cloning, expression and reconstitution of the trypanothione-dependent peroxidase system of Crithidia fasciculata. Mol. Biochem. Parasitol. **96**, 111–123 (1998). 9851611 10.1016/s0166-6851(98)00120-0

[28] K. G. Klein, C. L. Olson, J. E. Donelson, D. M. Engman, Molecular comparison of the mitochondrial and cytoplasmic hsp70 of *Trypanosoma cruzi*, *Trypanosoma brucei* and Leishmania major. J. Eukaryot. Microbiol. **42**, 473–476 (1995). 7581323 10.1111/j.1550-7408.1995.tb05893.x

[29] Q. Zhou, G. Dong, Z. Li, Flagellum inheritance in *Trypanosoma brucei* requires a kinetoplastid-specific protein phosphatase. J. Biol. Chem. **293**, 8508–8520 (2018). 29666191 10.1074/jbc.RA118.002106 PMC5986212

[30] T. An, Q. Zhou, H. Hu, H. Cormaty, Z. Li, FAZ27 cooperates with FLAM3 and ClpGM6 to maintain cell morphology in *Trypanosoma brucei*. J. Cell Sci. **133**, jcs245258 (2020). 32393602 10.1242/jcs.245258 PMC7295586

[31] F. Guegan , A long noncoding RNA promotes parasite differentiation in African trypanosomes. Sci. Adv. **8**, eabn2706 (2022). 35704590 10.1126/sciadv.abn2706 PMC9200285

[32] T. M. Stanne, M. Kushwaha, M. Wand, J. E. Taylor, G. Rudenko, TbISWI regulates multiple polymerase I (Pol I)-transcribed loci and is present at Pol II transcription boundaries in *Trypanosoma brucei*. Eukaryot. Cell **10**, 964–976 (2011). 21571922 10.1128/EC.05048-11 PMC3147422

[33] H. Reis, M. Schwebs, S. Dietz, C. J. Janzen, F. Butter, TelAP1 links telomere complexes with developmental expression site silencing in African trypanosomes. Nucleic Acids Res. **46**, 2820–2833 (2018). 29385523 10.1093/nar/gky028 PMC5888660

[34] A. Amiguet-Vercher , Loss of the mono-allelic control of the VSG expression sites during the development of *Trypanosoma brucei* in the bloodstream. Mol. Microbiol. **51**, 1577–1588 (2004). 15009886 10.1111/j.1365-2958.2003.03937.x

[35] J. R. Seed, S. J. Black, A proposed density-dependent model of long slender to short stumpy transformation in the African trypanosomes. J. Parasitol. **83**, 656–662 (1997). 9267408

[36] S. Trindade , *Trypanosoma brucei* parasites occupy and functionally adapt to the adipose tissue in mice. Cell Host Microbe. **19**, 837–848 (2016). 27237364 10.1016/j.chom.2016.05.002 PMC4906371

[37] P. Capewell , The skin is a significant but overlooked anatomical reservoir for vector-borne African trypanosomes. Elife **5**, e17716 (2016). 27653219 10.7554/eLife.17716 PMC5065312

[38] D. Mabille , Impact of pulmonary African trypanosomes on the immunology and function of the lung. Nat. Commun. **13**, 7083 (2022). 36400767 10.1038/s41467-022-34757-w PMC9674601

[39] S. Trindade , Slow growing behavior in African trypanosomes during adipose tissue colonization. Nat. Commun. **13**, 7548 (2022). 36481558 10.1038/s41467-022-34622-w PMC9732351

[40] A. K. Beaver , Extravascular spaces are the primary reservoir of antigenic diversity in *Trypanosoma brucei* infection. bioRxiv [Preprint] (2023). 10.1101/2022.06.27.497797 (Accessed 11 April 2023).

[41] S. Schuster , Unexpected plasticity in the life cycle of *Trypanosoma brucei*. Elife **10**, e66028 (2021). 34355698 10.7554/eLife.66028 PMC8448533

[42] M. Engstler, M. Boshart, Cold shock and regulation of surface protein trafficking convey sensitization to inducers of stage differentiation in *Trypanosoma brucei*. Genes. Dev. **18**, 2798–2811 (2004). 15545633 10.1101/gad.323404 PMC528899

[43] W. J. Herbert, W. H. Lumsden, *Trypanosoma brucei*: A rapid “matching” method for estimating the host’s parasitemia. Exp. Parasitol. **40**, 427–431 (1976). 976425 10.1016/0014-4894(76)90110-7

[44] Y. Hao , Integrated analysis of multimodal single-cell data. Cell **184**, 3573–3587.e29 (2021). 34062119 10.1016/j.cell.2021.04.048 PMC8238499

[45] J. Schindelin , Fiji: An open-source platform for biological-image analysis. Nat. Methods **9**, 676–682 (2012). 22743772 10.1038/nmeth.2019 PMC3855844

[46] S. M. Lanham, D. G. Godfrey, Isolation of salivarian trypanosomes from man and other mammals using DEAE-cellulose. Exp. Parasitol. **28**, 521–534 (1970). 4993889 10.1016/0014-4894(70)90120-7

[47] E. M. Briggs , Profiling the bloodstream form and procyclic form *Trypanosoma brucei* cell cycle using single cell transcriptomics. ELife **12**, e86325 (2023). 37166108 10.7554/eLife.86325 PMC10212563

[48] M. D. Young, S. Behjati, SoupX removes ambient RNA contamination from droplet-based single-cell RNA sequencing data. Gigascience **9**, giaa151 (2020). 33367645 10.1093/gigascience/giaa151 PMC7763177

[49] G. Finak , MAST: A flexible statistical framework for assessing transcriptional changes and characterizing heterogeneity in single-cell RNA sequencing data. Genome Biol. **16**, 278 (2015). 26653891 10.1186/s13059-015-0844-5 PMC4676162

[50] I. Tirosh , Dissecting the multicellular ecosystem of metastatic melanoma by single-cell RNA-seq. Science **352**, 189–196 (2016). 27124452 10.1126/science.aad0501 PMC4944528

[51] S. K. Archer, D. Inchaustegui, R. Queiroz, C. Clayton, The cell cycle regulated transcriptome of *Trypanosoma brucei*. PLoS One **6**, e18425 (2011). 21483801 10.1371/journal.pone.0018425 PMC3069104

[52] E. M. Briggs, When is a slender not a slender? scRNA-seq analysis of Trypanosoma brucei bloodstream forms from mammalian infection (1.0). Zenodo. 10.5281/zenodo.7778583. Deposited 3 April 2023.

[53] S. D. Larcombe, , scRNA-seq of Trypanosoma brucei from mouse infection, with and without Hyp2 RNAi induction. ENA. https://www.ebi.ac.uk/ena/browser/view/PRJEB60851?show=related-records. Deposited 6 April 2023.

