# Supplementary material for: The developmental hierarchy and scarcity of replicative slender trypanosomes in blood challenges their role in infection maintenance
Source: Proc Natl Acad Sci U S A. 2023 Oct 12;120(42):e2306848120. doi: 10.1073/pnas.2306848120 (PMC10589647; doi:10.1073/pnas.2306848120)

The developmental hierarchy and scarcity of proliferative slender parasites challenges the role of bloodstream trypanosomes in infection maintenance

Stephen D. Larcombe, Emma M. Briggs, Nick Savill, Balazs Szoor and Keith R. Matthews

Supplementary Figures

Supplementary Figure 1

Phase, DAPI and PAD1 staining for parasites isolated at 120h (a), 128h (b) and 152h (c), these being the source images for the merged panels shown in Figure 1e. Scale bar=10µm. Panel d shows representative images of fields of parasites from 120h, 128h and 456h post infection counterstained with DAPI (light blue) and PAD1 green. Individual parasites are highlighted in red boxes.

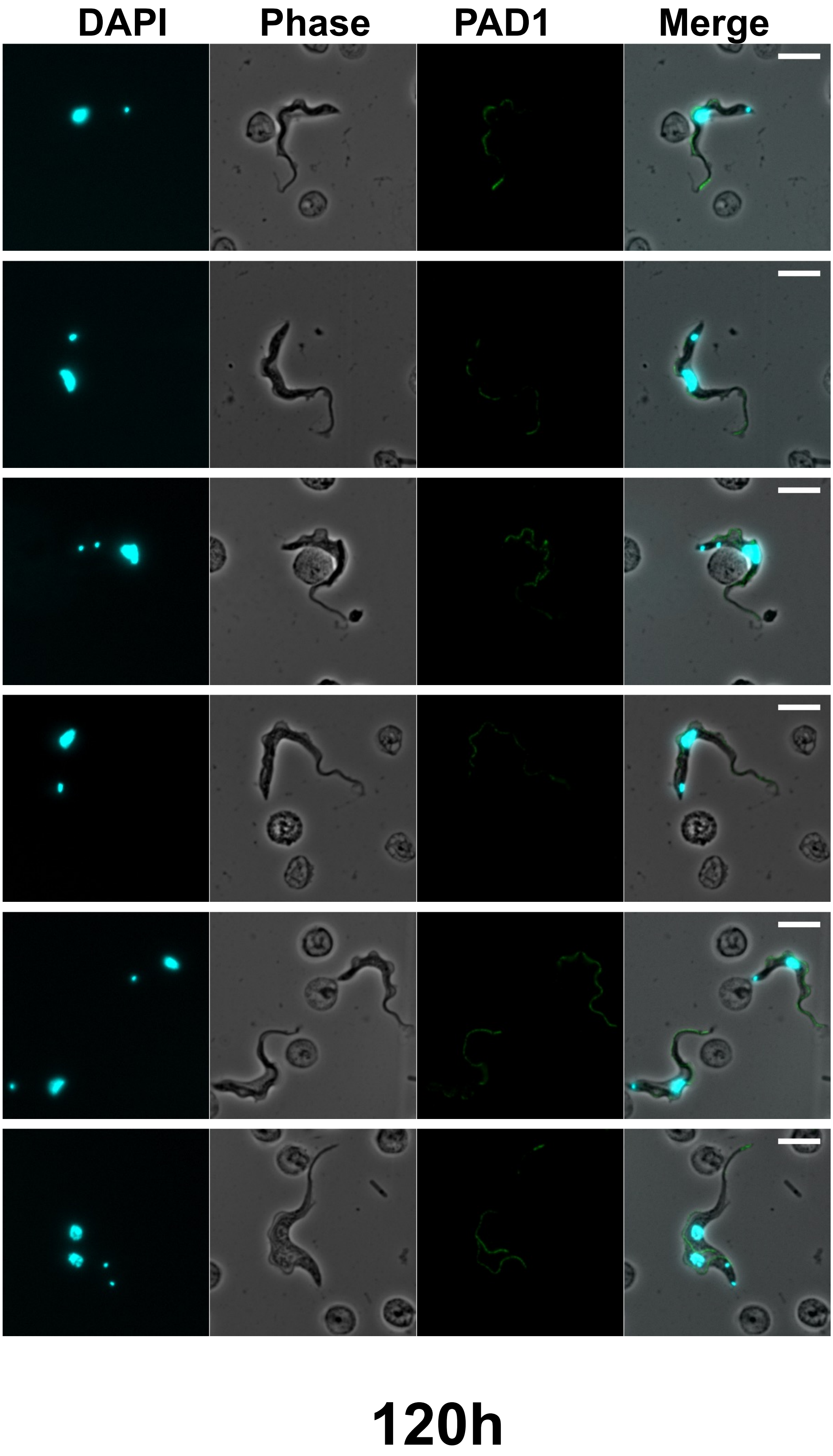

Supplementary Figure 1 A

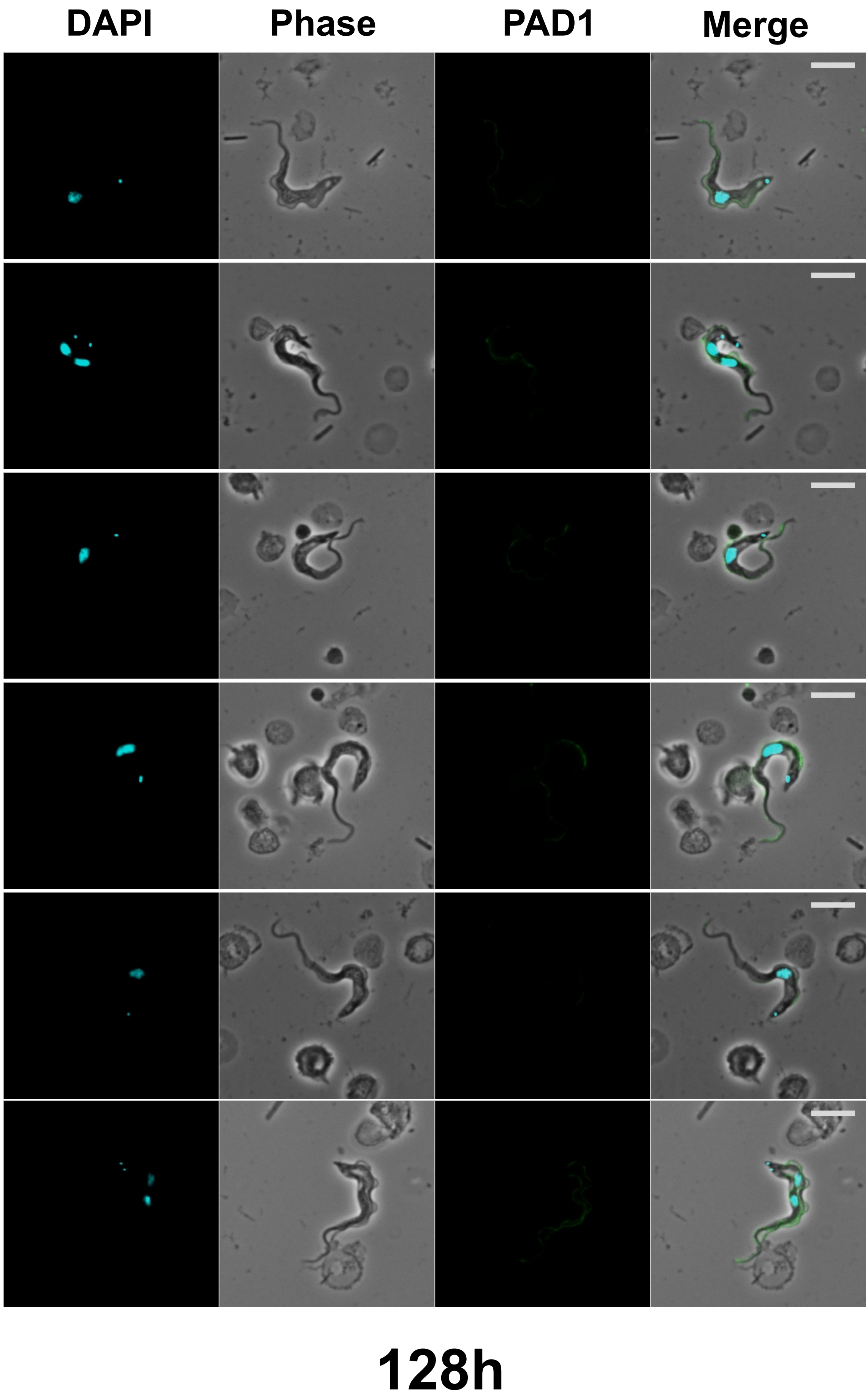

Supplementary Figure 1 B

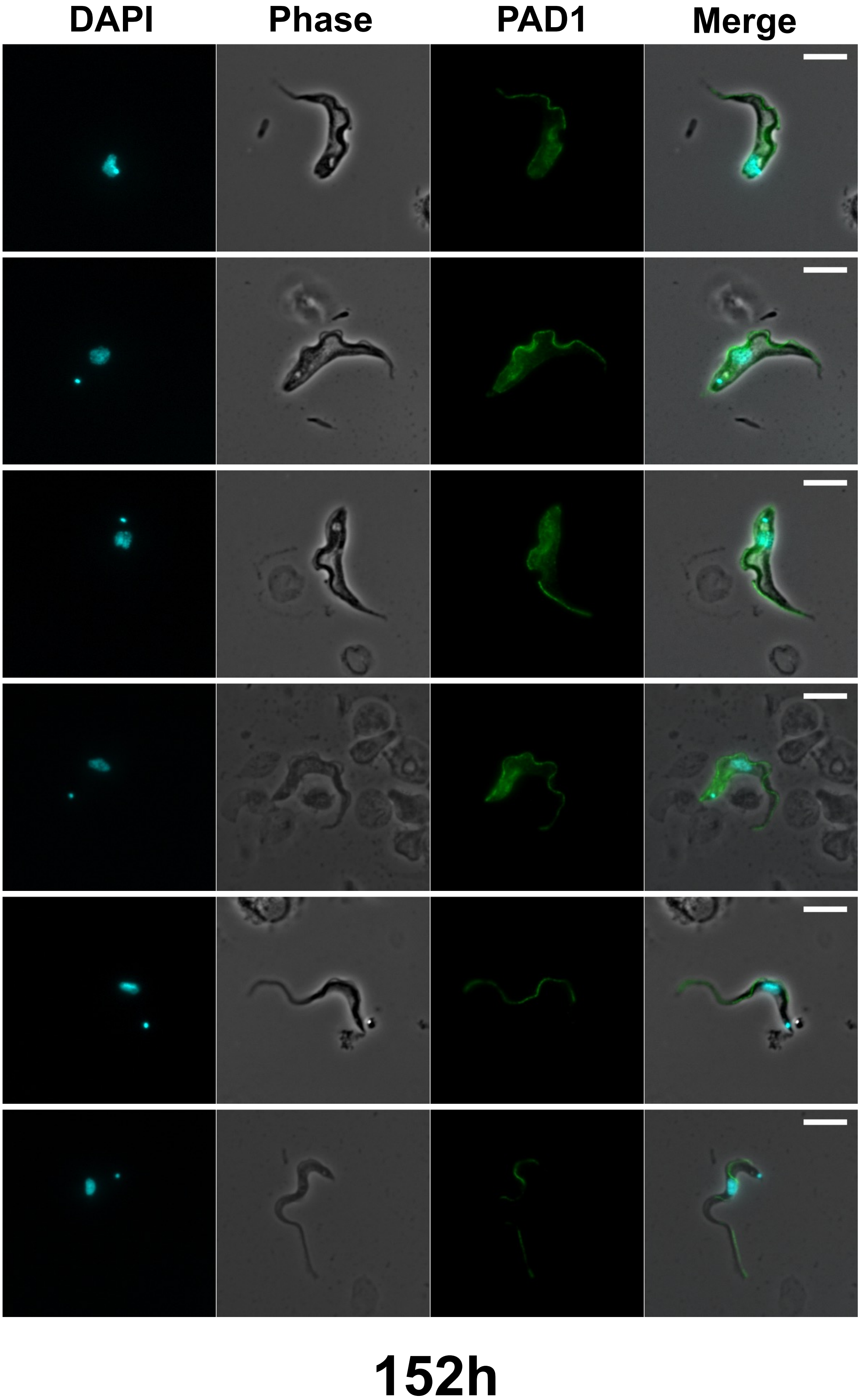

Supplementary Figure 1 C

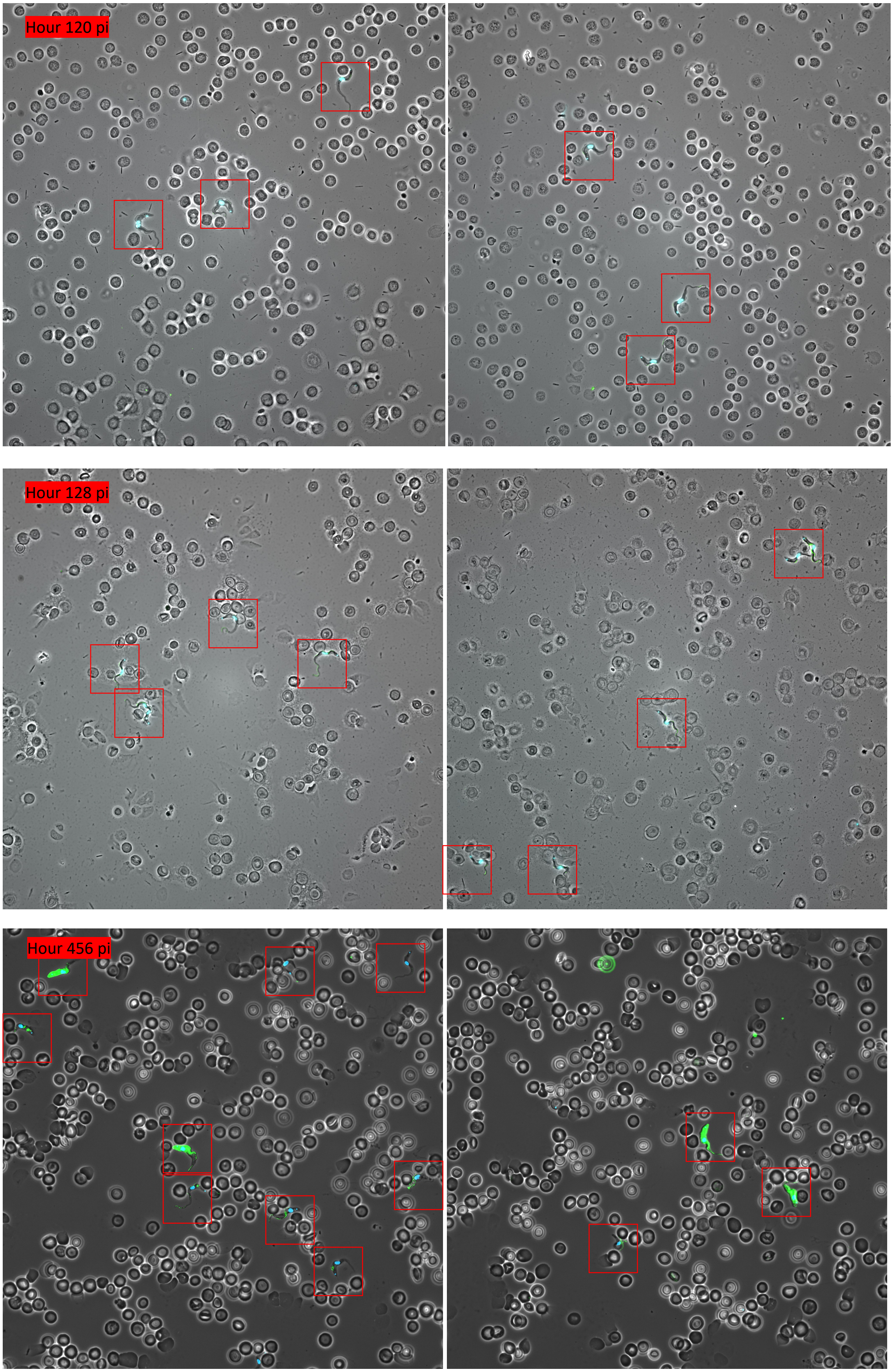

Supplementary Figure 1 D

Supplementary Figure 2

Metadata for the single cell RNA seq analysis of each sample (d7 +dox; d7 -dox; d23 +DOX; d23 -dox) before and after quality control filtering.

ScRNA-seq sample sumaries before and after quality control filtering

d7m = day 7 minus dox, d7p = day 7 plus dox, d23m = day 23 minus dox, day 23 p = day 23 plus dox. (dox = doxycline induction of Hyp2 RNAi)

sample	no. cell before QC	no. cell after QC	median UMI per cell	median features per cell	median % of transcrip	median % of transcripts encoding rRNA kDNA per cell
d7m	18079	3826	1577	1389	1.174394426	1.382273421
d7p	10715	7721	1607	1363	0.924811301	2.485421341
d23m	9719	8435	1629	1333	1.542475956	2.742761405
d23p	8732	7590	1810	1428	1.630880926	2.057249839

QC threshold used for each sample

sample	min. UMI	max. UMI	min. Feature	max. Feature	max. % kDNA	max. % rRNA
d7m	1000	2100	1000	1720	2	3
d7p	900	2700	850	2000	1.8	5
d23m	500	3000	550	2100	2.9	5.5
d23p	500	3200	550	2200	2.9	4.5

Number of unique UMI (unique transcripts) per cell. Red dashed line indicates QC thresholds

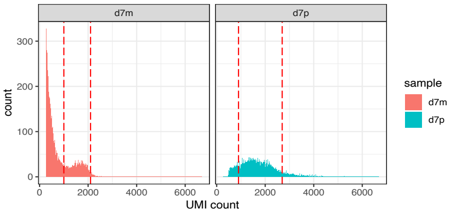

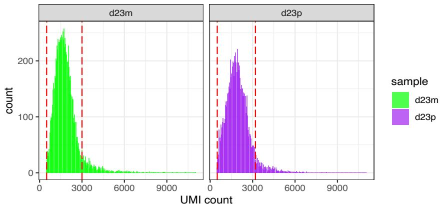

Number of unique features per cell. Red dashed line indicates QC thresholds

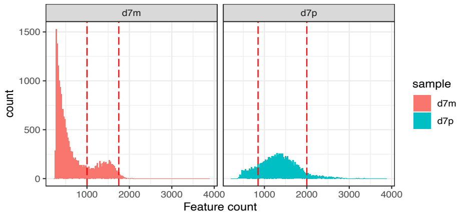

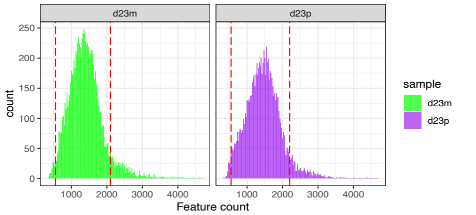

Percetage of transcripts per cell tgat are encoded on the kDNA maxicircle. Red dashed line indicates QC thresholds

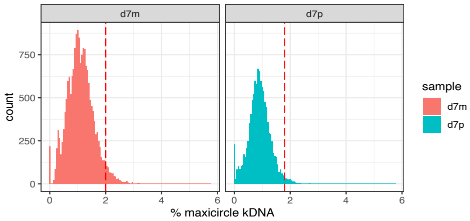

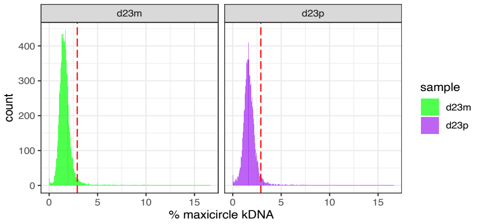

Percetage of transcripts per cell that encode ribosome RNA. Red dashed line indicates QC thresholds

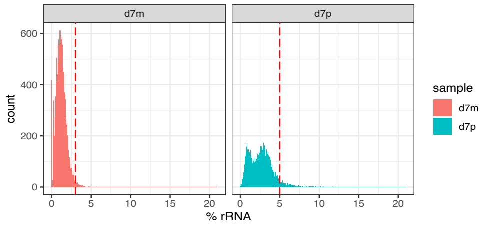

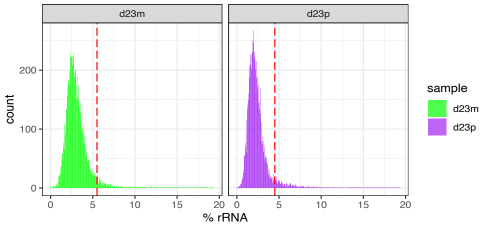

Supplementary Figure 3

scRNA-seq reveals the majority of blood form *T. brucei* lack expression of cell cycle phase marker genes. a) Average expression score of G1 (left), S (centre) and G2/M (right) phase marker genes for each parasite from all samples (D7 -dox and +dox, and D23 -dox and +dox). Parasites are split by clusters (0-4, x-axis). Red dashed line show threshold applied (0.05), to assign a phase to each parasite. Cells with expression below 0.05 in all case were marked "Unlabelled". b) UMAPs showing parasites for each sample (left to right), coloured by the assigned cell cycle phase. c) The percentage of cells in each cluster (x-axis) assigned in each cell cycle phase. d) The percentage of cells from each sample (x-axis), in each cell cycle phase (left bars), as well as the percentage of cells able to replicate when removed from host (i.e. replication competent cells) (dark grey, right bars).

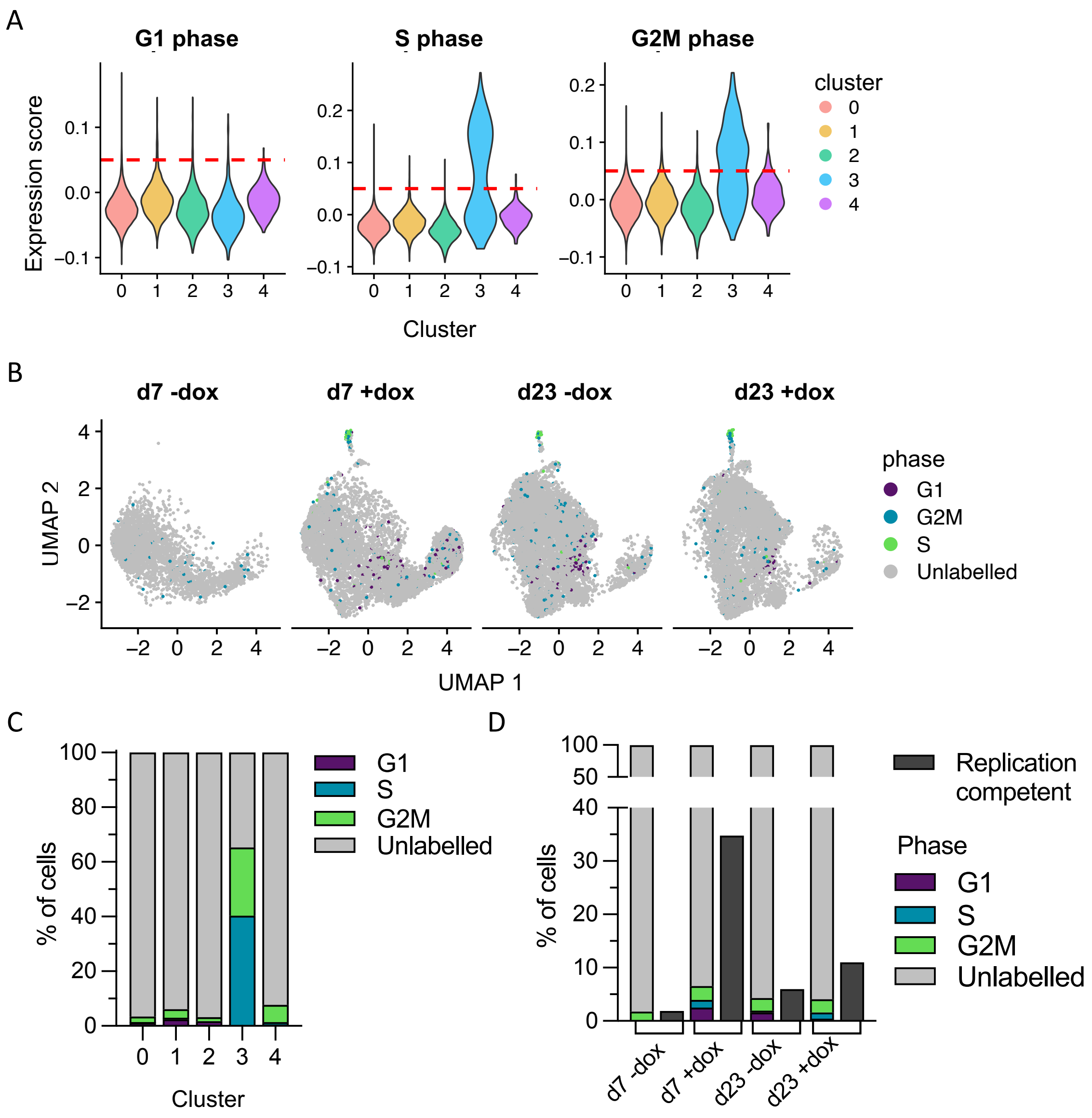

Supplement: Supplementary file 1 — Appendix 01 (PDF) [file pnas.2306848120.sapp.pdf]
